# De novo design and experimental characterization of bitter peptides

**DOI:** 10.1038/s41538-026-00942-0

**Published:** 2026-06-25

**Authors:** Alexandra Steuer, Francesco Ferri, Laura Eckrich, Julia Heidenkampf, Verena Karolin Mittermeier-Kleßinger, Silvia Schaefer, Maik Behrens, Noelia Ferruz, Corinna Dawid, Antonella Di Pizio

**Affiliations:** 1 Leibniz Institute for Food Systems Biology at the Technical University of Munich, Freising, Germany; 2 Professorship for Chemoinformatics and Protein Modelling, TUM School of Life Sciences, Technical University of Munich, Freising, Germany; 3 TUM School of Life Sciences Weihenstephan, Technical University of Munich, Freising, Germany; 4 Chair of Food Chemistry and Molecular Sensory Science, School of Life Sciences, Technical University of Munich, Freising, Germany; 5 Professorship for Chemosensory Food Systems, TUM School of Life Sciences, Technical University of Munich, Freising, Germany; 6 Centre for Genomic Regulation, The Barcelona Institute of Science and Technology, Barcelona, Spain; 7 Universitat Pompeu Fabra, Barcelona, Spain; 8 Atomistic Modeling Center, Munich Data Science Institute, Technical University of Munich, Garching, Germany

**Keywords:** Biochemistry, Biotechnology, Computational biology and bioinformatics, Drug discovery

## Abstract

Bitter taste is a critical quality determinant in food systems, particularly those using sustainable protein hydrolysates, where the unpredictable formation of bitter peptides severely limits consumer acceptance. Achieving predictive control over flavor chemistry requires deciphering the complex sequence-activity relationship. To address this, we integrated the generative capacity of a protein language model with BitterPep-GCN, a Graph Convolutional Network (GCN) capable of robust in silico bitter/non-bitter classification, to target the de novo design of functional bitter and non-bitter sequences. We achieved this by generating two strategic peptide libraries: a targeted tripeptide library derived from known bitter and non-bitter peptide sequences, and a set of de novo designed sequences. For the de novo designed peptides, we fine-tuned the conditional language model ZymCTRL on our curated dataset of sensory-validated bitter peptides (BPS-1000). Both libraries were subjected to classification and rigorous filtering using BitterPep-GCN to select high-confidence candidates for validation. The selected peptides were purchased and rigorously assessed for high purity. Sensory tests were conducted by an expert human panel to determine intrinsic taste quality and taste recognition thresholds. The results validated the high predictive fidelity of our pipeline: out of the 31 tested peptides, 25 were correctly classified, including 15 confirmed bitter and 10 confirmed non-bitter sequences. This study successfully demonstrates the application of machine learning frameworks in the design of bioactive peptides. It provides a set of novel taste-active peptides that can be used to accelerate the rational mitigation of off-tastes in next-generation food products.

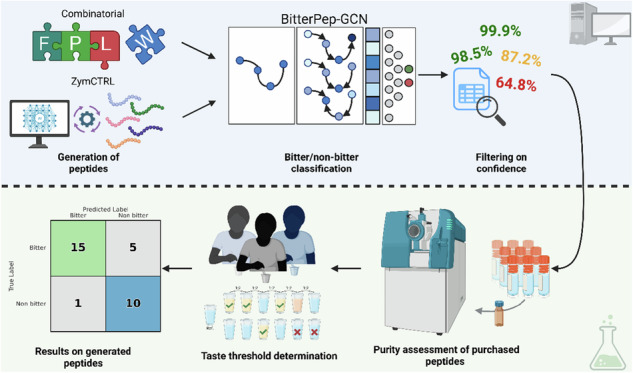

## Introduction

The bitter taste is one of the five basic taste modalities that influence our food choices^1^. It is a key factor in consumer acceptance, particularly in complex foods such as fermented products and protein hydrolysates^2,3^. Peptides derived from protein breakdown significantly contribute to this sensation and present a challenge for flavor scientists and food manufacturers seeking to control or eliminate off-tastes in functional and nutritional ingredients. This challenge has become critical as the food industry shifts toward sustainable, plant-based proteins. Processing these proteins often yields bitter peptides, which can severely hinder market viability^4^. To accelerate the adoption of these ingredients, we need to achieve predictive control over peptide formation. Accurate prediction of bitter peptides will enable the control of bitter peptide formation during processing, helping to tailor food production and ultimately improve the appeal of protein-rich foods.

Deciphering the complex relationship between a peptide’s amino acid sequence and its resulting bitter activity is an unresolved scientific challenge. Bitter peptides exhibit enormous structural variability, ranging widely in length, hydrophobicity, and physicochemical properties^5–10^. It is suggested that the bitter taste activity of peptides is mediated by a subset of bitter taste receptors (TAS2Rs)^11–13^. To facilitate research in this area, we have recently released a comprehensive database of experimentally validated bitter and non-bitter peptides, BPT-1000 (https://bps1000.leibniz-lsb.tum.de/), which includes sensory and receptor data. The database contains 570 bitter peptides and 423 non-bitter peptides, and illustrates the broad chemical space explored by bitter peptides^14^.

Machine Learning (ML) tools have become instrumental in navigating high-dimensional chemical space. ML models can enable the extraction of hidden, non-linear relationships, facilitating the rapid, high-throughput prediction of biological activity^15,16^. Building on these advances, we have developed a bitter peptide taste predictor, BitterPep-GCN, that decodes subtle sequence motifs using a graph-based representation of peptides^5^. This model provides robust in silico predictions of peptide bitter taste, and, due to its interpretable, graph-based architecture, it provides insights into sequence-activity relationships.

In this study, we developed and validated a machine learning framework for the design of functional bitter taste peptides, bridging sequence generation with experimental sensory validation to advance predictive flavor chemistry.

## Results and discussion

To demonstrate the applicability of our developed machine learning framework and explore the structural determinants of peptide bitterness, we designed two distinct peptide libraries with complementary objectives. The first library was constructed by systematically decomposing longer sequences into a comprehensive tripeptide dataset enriched for the chemical space of BPS-1000^14^. Building on our previous findings regarding the high sensory relevance of short peptide fragments^5^, this approach aimed to isolate the minimal structural units capable of maintaining bitter taste activity. The second library utilized the generative architecture of ZymCTRL^17^ to sample de novo sequences from scratch, testing the pipeline’s capacity to design functional entities independent of pre-existing templates. The taste activity predicted by BitterPep-GCN for both libraries was subsequently validated and quantified through human sensory experiments. The following sections present this integrated protocol, demonstrating its predictive fidelity and expanding the known molecular space of bitter peptides by identifying novel functional taste entities.

The underlying idea of this work, that bitter taste signatures could be identified as peptide motifs in longer bitter peptides, is based on the structural constraints inherent to TAS2R receptors. Due to the relatively constrained structure of their binding pockets, we hypothesized that receptor binding must be governed by specific, low-molecular-weight core motifs as hotspots, even for larger peptides. Our prior work on known bitter peptides (chemical space of BPS-1000) identified dipeptides as the most abundant structural unit within the known bitter peptide space^14^. However, receptor data show that the tripeptide WWW is one of the most potent bitter agonists known in receptor assays^11,18^. This emphasizes the necessity of systematically exploring the chemical space of tripeptides to fully capture the range of high-potency binders.

First, we sought to bridge the gap between in vitro receptor activity and human perception by selecting five TAS2R peptide agonists (FW, WF, WL, WP, and WWW) previously identified by Kohl et al.^11^ for sensory validation of their predicted bitterness in humans. Sensory tests confirmed all five peptides as bitter agonists, establishing the biological relevance of the previous receptor data. Crucially, the tripeptide WWW exhibited the highest potency with the lowest sensory threshold recorded at 4.1 µM (Table 1).

**Table 1:** Sensory evaluation of TAS2R peptide agonists, including the bitter taste thresholds (TT)

Peptide	Taste qualities (number of panelists)	Bitter TT [µmol/L]^1^	Bitter TT [mg/L]^1^
FW	bitter (11), sweet (5), astringent (4), sour (2), salty (1)	623	219
WF	bitter (12), astringent (5), sour (1)	102	35.7
WL	bitter (12), astringent (6), sour (2)	403	128
WP	bitter (11), sour (2)	519	161
WWW	bitter (12), astringent (4), sour (3), sweet (2)	4.13	2.38

Given the high potency of the tripeptide WWW and the fact that most previous sensory analyses primarily focused on dipeptides, we decided to perform a comprehensive in silico screen of the tripeptide chemical space to expand our molecular understanding of this size of peptides. We started by processing all longer peptides (i.e., those with four or more residues) in BPS-1000, the current state-of-the-art database of bitter peptide knowledge^14^. We performed an exhaustive, sequential decomposition of every longer peptide into its constituent three-residue fragments. This process involves sequentially cleaving the peptide after the third residue, then moving one residue and repeating the cleavage until the end of the chain is reached. For instance, a hexapeptide (R_1_R_2_R_3_R_4_R_5_R_6_) yields four overlapping tripeptides: (R_1_R_2_R_3_), (R_2_R_3_R_4_), (R_3_R_4_R_5_), and (R_4_R_5_R_6_). By systematically applying this method to both bitter and non-bitter entries in the database, we generated a total of 480 unique tripeptides that had not been previously reported or tested experimentally. Then we used the BitterPep-GCN predictor to assess the bitterness probability for every combination derived from this pool. The tripeptide dataset was classified into 191 tripeptides predicted to be bitter and 289 predicted to be non-bitter, providing a rich, targeted resource for bitter peptide identification.

**Fig. 1:**
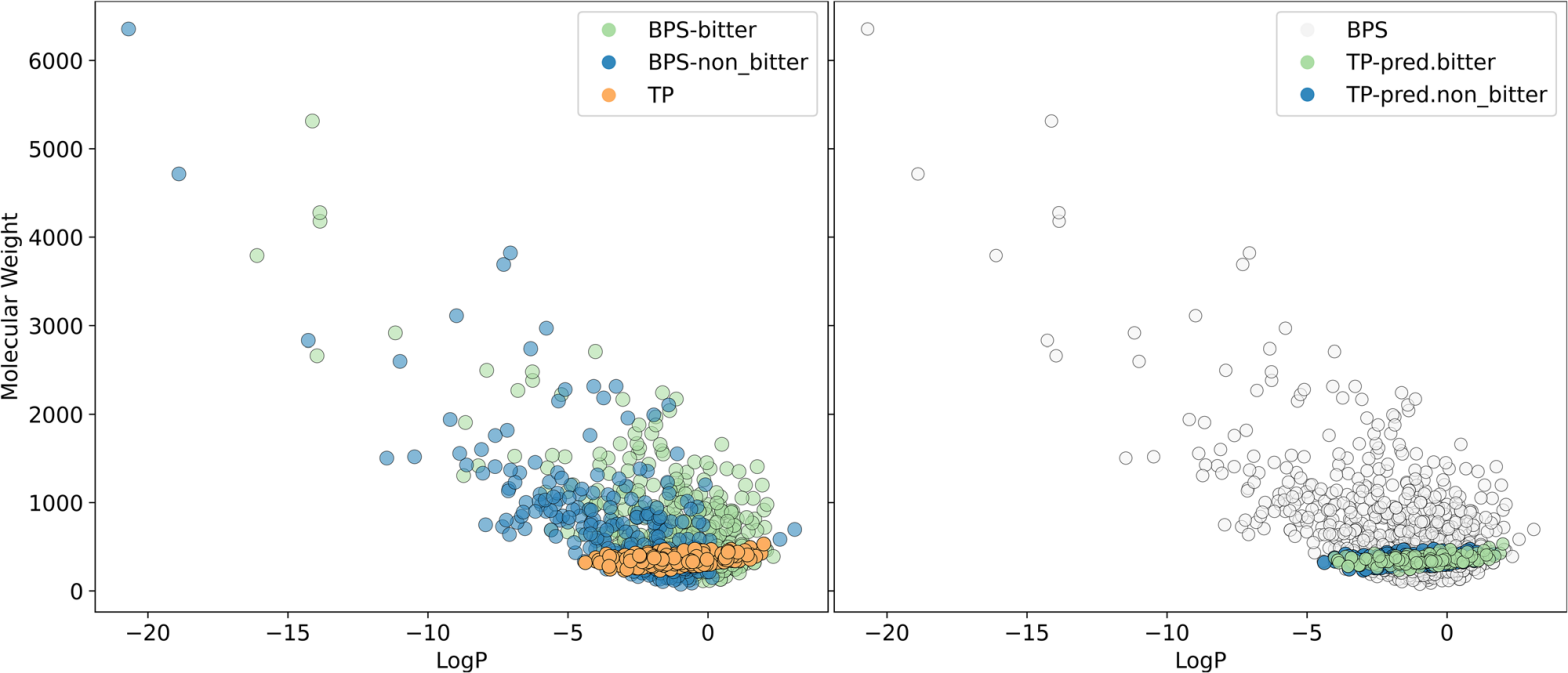
Chemical space of the generated tripeptide (TP) dataset. Tripeptides are represented as orange dots as projected on the BPS-1000 molecular space in green for bitter peptides and in blue for non-bitter peptides (left). The BPS-1000 space is colored in light grey in the background, and TP are colored according to bitter taste prediction (right).

Figure 1 shows the chemical space of BPS-1000, which is defined by hydrophobicity (LogP) *versus* size (molecular weight, MW). All tripeptides cluster within a similar MW range (Fig. 1A), from 231 Da for the tripeptide with the lowest MW (VGG) to 530 Da for the tripeptide with the highest MW (WYY). Although hydrophobicity has been revealed to be a central property of bitter-tasting peptides and a measure to distinguish between bitter and non-bitter peptides^19^, all tripeptides span an area of LogP ranging from approximately −5 to 0, and no significant differences are observed between predicted bitter and non-bitter peptides (Fig. 1B).

Peptides occupy a high-dimensional chemical space, with the number of possible sequences growing exponentially with chain length. Current knowledge of the bitter peptide space, as represented by the BPS-1000 database, covers a small portion of this potential space. Our previous approach of systematically generating tripeptides from BPS-1000 sequences successfully demonstrated the potential for expanding this knowledge base through simple sequence reassembly. We hypothesized that it is possible to decipher the underlying functional language of taste-active peptides to design entirely novel sequences with targeted bitter or non-bitter activity from scratch. This has become achievable through computational de novo sequence generation. Recent breakthroughs in ML-driven protein design have made this objective achievable by treating amino acid sequences as a functional language, a paradigm shift recognized by the 2024 Nobel Prize in Chemistry^20^. In this study, we translate this powerful generative principle from structural biology to flavor chemistry, establishing a pipeline for the design and computational-to-sensory validation of novel bitter taste-active peptides.

**Fig. 2:**
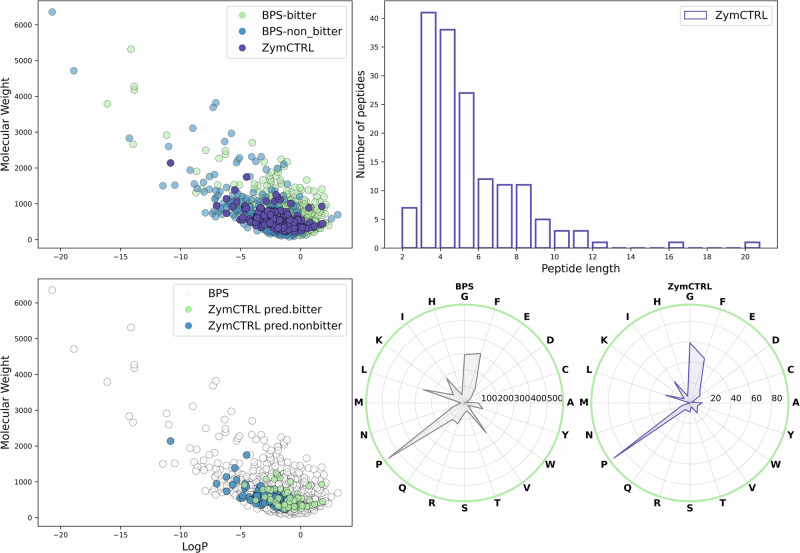
Chemical space and amino acid composition of de novo designed peptides. De novo designed peptides (DP), colored in purple, projected on the BPS-1000 (BPS) chemical space (top-left). Bitter BPS peptides are colored in green, while non-bitter peptides are colored in blue. Chemical space of the de novo designed peptides (ZymCTRL) colored by bitter prediction (green for bitter prediction and blue for non-bitter prediction) projected in the chemical space of the BPS-1000 (BPS), colored in light grey (bottom-left). Histogram representing the distribution of peptide length (top-right). Radar charts comparing the amino acid composition (total counts) of the BPS-1000 bitter peptides (grey) and the predicted bitter peptides generated by the ZymCTRL language model (violet) (bottom-right).

Acknowledging the limited size of the available bitter taste dataset, we employed Transfer Learning. The application involves two steps: pre-training and fine-tuning. During pre-training, the model learns rich, generalized amino acid features from large fundamental protein databases. In the fine-tuning step, these features are transferred and adapted for our smaller, bitter-taste-specific dataset. We selected the ZymCTRL generative language model^17^. ZymCTRL enables the generation of peptides that follow a learned probability distribution that reflects the desired properties present in a given dataset. By sampling sequences from a space informed by stable, biologically active enzymes, it increases the likelihood that the generated short peptides are both stable and naturally occurring, avoiding the generation of purely random or unstable sequences. Despite being trained on enzymes, ZymCTRL’s internal transformer architecture, specifically its attention mechanism, is highly effective at capturing sequence motifs, which are often responsible for high potency. Using this approach, we successfully compiled a novel set of 161 unique peptides. Projecting onto the BPS-1000 database confirmed that the generated peptides are entirely within the known bitter chemical space (Fig. 2A). We applied our BitterPep-GCN predictor to the newly generated library, which classified the new sequences as 89 predicted bitter peptides and 72 predicted non-bitter peptides (Fig. 2B). Interestingly, the predicted bitter peptides cluster in an area of higher LogP values, while the predicted non-bitter peptides tend to have lower LogP values and higher MW.

Although ZymCTRL was trained on sequences of various lengths, the generated dataset primarily includes peptides of up to nine amino acids, with a pronounced enrichment of tripeptides (Fig. 2C). This distribution aligns with our previous observations regarding the high sensory relevance of short chain lengths for bitter taste activity, despite tripeptides not being dominantly represented in the BPS-1000 dataset. As previously mentioned, BPS-1000 is instead imbalanced toward dipeptides. Interestingly, the amino acid composition of ZymCTRL-generated peptides is very similar to that of the BPS (Fig. 2D). Zooming-in on the bitter set, which is the one used to train ZymCTRL, the amino acids P, G, and F are the most frequent amino acids in bitter peptides within both the BPS-1000 and the ZymCTRL datasets (Fig. 2D).

To validate the predictive performance of our computational pipeline and test its ability to detect bitter peptide signatures, we selected a subset of predicted bitter and non-bitter peptides for sensory validation. Specifically, 30 peptides (Table 2) were selected, comprising 14 predicted bitter peptides (EVF, FFR, FPE, FRP, LEQ, PEV, PNS, RPP, RRP, LDL, RPY, GDFF, WGG, GPPG) and 16 candidate non-bitter peptides (AME, EVM, PQN, PVR, QPE, QQK, QQP, QTP, RPL, RAPF, RQPF, KALPQ, KAP, KLP, KI, VAEQ). Peptides were selected to represent a diverse cross-section of the predicted chemical space while prioritizing sequences with high-confidence scores from BitterPep-GCN. The identity and purity of all purchased peptides were meticulously verified prior to sensory analysis using UHPLC-ToF-MS and qHNMR^21^.

**Table 2:** Sensory validation of selected peptides

Peptide	Dataset	BitterPep-GCN prediction	Taste qualities (number of panelists)	Bitter TT [µmol/L]	Bitter TT [mg/L]
**EVF**	TP	bitter	bitter (11), sour (11), astringent (4), sweet (3), salty (2)	259	102
**FFR**	TP	bitter	bitter (12), astringent (6), sweet (1)	105	49.4
**FPE**	TP	bitter	bitter (12), sour (7), astringent (3), sweet (1), salty (1)	851	333
**FRP**	TP	bitter	bitter (11), astringent (6), sour (1)	285	112
**LEQ**	TP	bitter	sour (10), bitter (9), astringent (2), salty (1)	237	91.9
**PEV**	TP	bitter	bitter (10), sour (10), astringent (2), sweet (2), salty (1)	153	52.5
**PNS**	TP	bitter	bitter (9), astringent (4), umami (2), sour (2)	342	108
**RPP**	TP	bitter	bitter (8), astringent (4), sour (3), sweet (1)	297	109
**RRP**	TP	bitter	bitter (9), astringent (5), sour (1), umami (1)	340	145
**AME**	TP	non-bitter	sour (12), bitter (3), sweet (2), astringent (2), salty (1), umami (1)		
**EVM**	TP	non-bitter	sour (12), sweet (3), astringent (2), umami (2), bitter (2)		
**PQN**	TP	non-bitter	sour (12), astringent (2), sweet (1), bitter (1)		
**PVR**	TP	non-bitter	sour (11), astringent (3), sweet (2), bitter (2)		
**QPE**	TP	non-bitter	sour (9), astringent (6), bitter (5), salty (2), umami (1)		
**QQK**	TP	non-bitter	sour (11), bitter (4), astringent (3), sweet (2), salty (1)		
**QQP**	TP	non-bitter	sour (8), astringent (5), bitter (3), sweet (2)		
**QTP**	TP	non-bitter	sour (5), bitter (3), astringent (2), sweet (2), salty (1)		
**RPL**	TP	bitter	bitter (8), astringent (7), sweet (1), sour (1), umami (1)	499	210
**RAPF**	ZymCTRL	bitter	bitter (8), sweet (2), astringent (2), sour (1)	397	252
**RQPF**	ZymCTRL	bitter	bitter (7), astringent (4), sour (2)	1093	217
**KAP**	ZymCTRL	non-bitter	bitter (4), sour (4), astringent (3), sweet (1)	793	249
**KLP**	ZymCTRL	bitter	bitter (9), sour (5), astringent (3)	435	155
**GDFF**	ZymCTRL	bitter	astringent (5), bitter (4), salty (2), sour (1), sweet (1)	435	264
**GPPG**	ZymCTRL	bitter	bitter (7), astringent (3), sour (1), sweet (1)	547	224
**VAEQ**	ZymCTRL	non-bitter	sour (8), bitter (2), astringent (2), salty (1)	1059	
**TAEQ**	ZymCTRL	non-bitter	bitter (7), astringent (4), sour (3), sweet (1)	688	195
**LDL**	ZymCTRL	bitter	bitter (5), sour (5), astringent (3), sweet (1)	546	294
**RPY**	ZymCTRL	bitter	bitter (5), sour (4), astringent (4), sweet (2), salty (1), umami (1)	818	217
**KALPQ**	ZymCTRL	non-bitter	bitter (5), salty (3), sweet (2), astringent (2)	515	588
**KI**	ZymCTRL	bitter	bitter (6), astringent (4), sour (3), sweet (1)	533	138

The columns include: the peptide sequence; the dataset source (indicating if the peptide originated from the systematic decomposition of the BPS-1000 tripeptide library [TP] or was generated by ZymCTRL); the BitterPep-GCN classification; the resulting taste qualities reported by the panelists (with the number of panelists in parentheses); and the experimental Bitter Taste Thresholds (Bitter TT) in both µmol/L and mg/L.

The screening and sensory evaluation allowed us to validate our in silico predictions and identify novel bitter peptides. For the purpose of this study, a peptide was classified as bitter if the bitter taste was detected by at least 7 of the 12 panelists (*n* ≥ 7), and classified as non-bitter if the bitter taste was detected by no more than 4 panelists (*n* ≤ 4). Based on these criteria, the sensory analysis confirmed the identification of 15 novel bitter peptides and 11 non-bitter peptides. The remaining four peptides (LDL, RPY, KALPQ, and KI) were inconclusive, as the panel consensus for bitter taste detection fell between the threshold range (i.e., detected by 5 or 6 panelists). Additionally, non-bitter taste attributes (e.g., sourness, saltiness) were reported by a small number of panelists (*n* ≤ 6) for several peptides but were not deemed statistically significant across the entire tested concentration range. However, four of the confirmed bitter peptides (EVF, FPE, LEQ, and PEV) were not only perceived as bitter but also demonstrated an additional sour taste attribute at higher concentrations, with distinct sour taste thresholds also documented (Table 2). This co-occurrence of sourness may be attributed to the peptide’s intrinsic sour taste or its potential to modulate sour taste receptors at the testing concentration and in the standardized sensory testing protocol, where solutions are adjusted to a slightly acidic pH to ensure peptide stability and mimic physiological or food conditions. Specifically, the presence of Glutamic acid (E) in such sequences (EVF, FPE, LEQ, and PEV) likely contributes to this perceived sourness, as this acidic residue is a recognized sour-taste determinant^22^.

**Fig. 3:**
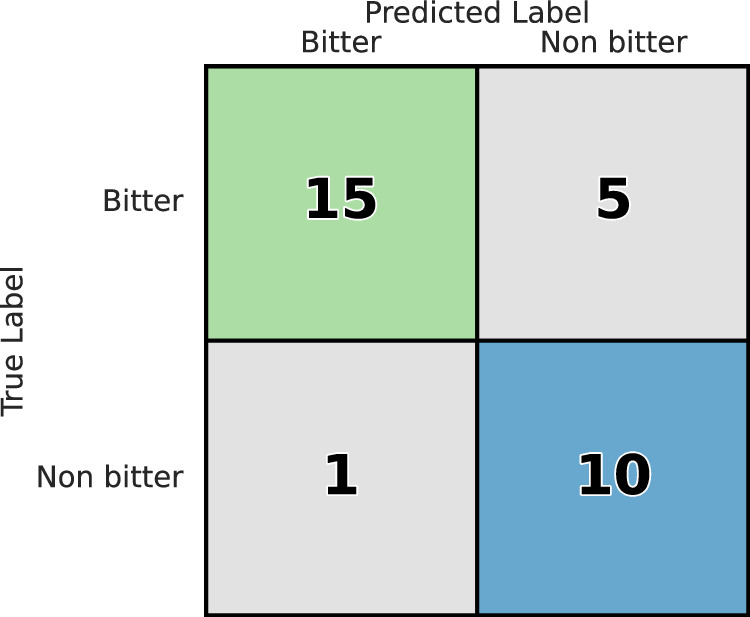
Confusion matrix of the BitterPep-GCN model performance on the total of the 31 peptides, including 26 peptides reported in Table 2, excluding the 4 peptides without consensus, and the selected peptides for human sensory tests reported in Table 1. Both these sets of peptides are external to the training set of the BitterPep-GCN model.

Of the candidates tested, 15 predicted bitter peptides and 10 predicted non-bitter peptides were correctly identified (Fig. 3), resulting in a predictive accuracy of 80% across the validated subset (*n* = 31). To further characterize performance, we calculated a precision of 93% and an F1-score of 0.83. Even when adopting a conservative approach, treating the four inconclusive peptides as incorrect predictions, the model maintains a high degree of reliability (Fig. S4). These findings validate our integrated computational-to-sensory protocol as a robust framework for the de novo design and experimental discovery of functional, taste-active peptides.

In summary, this study is a significant advancement in predictive flavor chemistry as it shifts the focus from prediction to peptide design. While several machine learning tools have been developed for bitter peptide prediction^8,10^, this work provides experimental sensory validation of a series of designed peptides. With 25 out of 31 tested peptides correctly classified, the high accuracy of our pipeline demonstrates the real-world applicability of the BitterPep-GCN model. The novel peptides identified not only prove the workflow’s predictive ability but also provide an expanded understanding of bitter peptides (Table S1 and Figs. S1–3**)**. These results prove that bitter taste can be predicted and re-engineered, providing novel molecular insights to mitigate off-flavors in next-generation food products.

## Methods

The computational analysis and modeling of the peptides were performed using KNIME (Konstanz Information Miner), an open-source platform for data processing and analysis (https://www.knime.com). Specialized KNIME extensions were integrated for individual functional steps, alongside a Python environment for data visualization and customized evaluations. Leveraging these tools, we established a centralized, custom KNIME workflow. To ensure reproducibility and open-science compliance, this workflow, along with the curated dataset, models, and generated peptide sequences, has been made publicly available on our GitHub repository (https://github.com/DiPizio-Lab/bitter-peptide-design). The individual methodological components of this integrated pipeline are detailed below.

### Exhaustive generation of tripeptides

The tripeptide dataset was generated by systematically decomposing longer peptides from the BPS-1000 database^14^. The BPS-1000 database contains a comprehensive collection of both bitter and non-bitter peptides, and only sequences with a length *L* ≥ 4 amino acid residues were processed for fragmentation. Peptide decomposition was performed using a sliding window approach of size *n* = 3 residues to generate every possible, overlapping tripeptide motif. Starting from the N-terminus, the first tripeptide was extracted (residues R_1_R_2_R_3_). The window was then shifted by a single residue, extracting the next tripeptide (residues R_2_R_3_R_4_), and the process was repeated until the window reached the C-terminus. This systematic process ensures that all tripeptide sequences contained within the longer peptides are captured.

### De novo peptide generation

For the generation of bitter peptides, we used ZymCTRL, a conditional language model originally developed for the de novo generation of artificial enzymes belonging to specific Enzyme Commission (EC) classes within the BRENDA database^17^. For fine-tuning, the pre-trained ZymCTRL model weights were updated using sensory-validated bitter peptides. The training data was extracted from the BPS-1000 database (accessed: 21.09.2024). A rigorous curation step was applied to exclude peptides with inconsistent or mixed taste quality reports, resulting in a final bitter peptide dataset of 478 amino acids and peptide sequences. To condition the generation specifically toward the bitter attribute, a synthetic EC number, 1.2.3.16 (unassigned in BRENDA), was arbitrarily chosen and assigned to all bitter sequences in the dataset. The curated dataset was randomly partitioned into a training set (50%) and an evaluation set (50%). The hyperparameters were chosen based on the proposal at Huggingface (training epochs = 28, learning rate = 0.8e−04). We followed the exact protocol outlined in the original ZymCTRL methodology, specifically “Example 2: Fine-tuning on a set of user-defined sequences,” available via the Huggingface repository (https://huggingface.co/AI4PD/ZymCTRL). Following fine-tuning, the model was employed to generate 1504 novel peptide sequences conditioned on the assigned EC number (1.2.3.16). The generated sequences were subjected to post-processing filters to ensure novelty and remove duplicate entries.

### BitterPep-GCN predictions

To classify the bitter activity of the generated tripeptides and de novo generated peptides, the BitterPep-GCN model was used. This model is a Graph Convolutional Network specifically optimized for the binary classification of bitter and non-bitter peptides. BitterPep-GCN was originally trained on the BTP640 benchmark dataset. The default hyperparameters from the published study were retained for this application. The peptide sequences were processed into a graph-based data structure. Each amino acid residue was treated as a node, and the covalent peptide bonds linking the residues were represented as edges, thereby capturing the topological relationships essential for GCN processing.

### Physiochemical and molecular descriptors

The solubility of all predicted peptides in water was estimated using the web-based tool PepCalc Peptide Solubility Calculator (available at https://pepcalc.com/peptide-solubility-calculator.php). Both the SLogP (octanol-water partition coefficient) and Molecular Weight (MW) calculations were performed using the RDKit Python library, specifically employing the rd.chem module.descriptors module within the KNIME workflow environment^23,24^.

### Human sensory tests

Peptide agonists of TAS2Rs (Table 1) and a subset of 30 peptides (Table 2) from the generated tripeptide and ZymCTRL libraries were selected for human sensory validation. The selection of the in silico-generated peptides was based on high-confidence predictions from the BitterPep-GCN model and calculated physicochemical properties, with an emphasis on motifs that occurred within the comprehensive BPS-1000 database (for the tripeptide library). Sensory sessions for the selection of the dataset were performed in single sensory booths in an air-conditioned room at 22–25 °C. A sensory panel of 12 experienced human assessors (aged 23–49 years) evaluated all peptides except RQPF and VAEQ, for which only 11 panelists were available. Panelists had given informed consent to participate in the sensory tests of the present investigation and had no history of known taste disorders. To avoid retronasal interactions, nose clips were worn for every sensory session. The panelists rinsed their mouths with commercially available bottled water (Evian, Évian-les-Bains, France) with low mineral content before and between each sensory sample. To become familiar with the taste language and impressions, the panelists were trained to identify and evaluate aqueous supra-threshold reference solutions with bitter, salty, sour, sweet, astringent, umami, kokumi active compounds as reported earlier^25–27^: caffeine (2 mmol/L) for bitter taste, NaCl (20 mmol/L) for salty taste, D sucrose (20 mmol/L) for sweet taste, L-lactic acid (20 mmol/L) for sour taste, monosodium L glutamate (6 mmol/L) for umami taste, tannic acid (0.05%) for astringent sensation, and model broth spiked with reduced glutathione for kokumi activity.

### Human taste recognition threshold

The intrinsic taste quality and threshold concentrations of the peptides were determined by means of duo-trio-tests. The experiments were approved by the ethics commission of the Technical University of Munich, Faculty for Medicine (reference number 449/20 S-EB). The study was performed in accordance with the Declaration of Helsinki. The peptides were dissolved in bottled water adjusted to pH 5.5 with trace amounts of formic acid and diluted 1:2, except for GDFF. The sensory panel tasted the dilution series, starting with the lowest concentration against a blank sample (bottled water with pH 5.5). The peptide GDFF was not soluble at pH 5.5. To achieve solubility, the pH had to be adjusted to 6.1. Therefore, this peptide was tested at pH 6.1. Evian water adjusted to pH 6.1 was used as a reference for dilutions and as the “negative” sample in the duo-trio tests. The intrinsic taste recognition threshold of every panelist was calculated as the geometric mean of the first missed and the last correctly identified concentration. The geometric mean of the individual threshold concentrations of all panelists was calculated and represents the intrinsic taste recognition threshold of the sensory panel.

## Supplementary information

Supplementary Information

## Data Availability

All data supporting the findings of this study are available within the article and its Supporting Information file. The complete datasets, curated ligand lists, computational models, and the specific KNIME workflows developed and utilized in this work have been deposited in a public repository and can be fully accessed via GitHub at https://github.com/DiPizio-Lab/bitter-peptide-design.
