# Supplementary material for: De novo design and experimental characterization of bitter peptides
Source: NPJ Sci Food. 2026 Jun 25;10:200. doi: 10.1038/s41538-026-00942-0 (PMC13303823; doi:10.1038/s41538-026-00942-0)
Supplement: Supplementary file 1 — Supplementary Information [file 41538_2026_942_MOESM1_ESM.pdf]

Supporting Information

De Novo Design and Experimental Characterization of Bitter Peptides

Alexandra Steuer[1,2,3], Francesco Ferri[1,2,3], Laura Eckrich[4], Julia Heidenkampf[5], Verena Karolin Mittermeier-Kleßinger[5], Silvia Schaefer[1,3], Maik Behrens[1], Noelia Ferruz[6,7], Corinna Dawid[1,4,5], Antonella Di Pizio[1,2,8]*

[1] Leibniz Institute for Food Systems Biology at the Technical University of Munich, 85354 Freising, Germany.

[2] Professorship for Chemoinformatics and Protein Modelling, School of Life Science, Technical University of Munich, 85354 Freising, Germany.

[3] TUM School of Life Sciences Weihenstephan, Technical University of Munich, Alte Akademie 8, 85354, Freising, Germany

[4] Chair of Food Chemistry and Molecular Sensory Science, School of Life Sciences, Technical University of Munich, 85354 Freising, Germany

[5] Professorship for Chemosensory Food Systems, School of Life Sciences, Technical University of Munich, 85354 Freising, Germany

[6] Centre for Genomic Regulation, the Barcelona Institute of Science and Technology, Dr Aiguader 88, Barcelona 08003, Spain

[7] Universitat Pompeu Fabra, Barcelona 08003, Spain

[8]Atomistic Modeling Center, Munich Data Science Institute, Technical University of Munich, Garching, Germany

*correspondance to: a.dipizio.leibniz-lsb@tum.de

	n	mean	std	median	min	max
ZymCTRL	161	-2.02	1.79	-1.95	-10.84	1.84
BPS-1000	780	-1.84	2.62	-1.35	-20.69	3.1
TP	450	-1.17	1.26	-1.14	-4.38	1.99

	n	mean	std	median	min	max
ZymCTRL	161	567.25	279.97	492.17	220.09	2140.06
BPS-1000	780	693.75	640.68	484.26	75.03	6357.26
TP	450	355.98	44.75	355.67	231.12	530.22

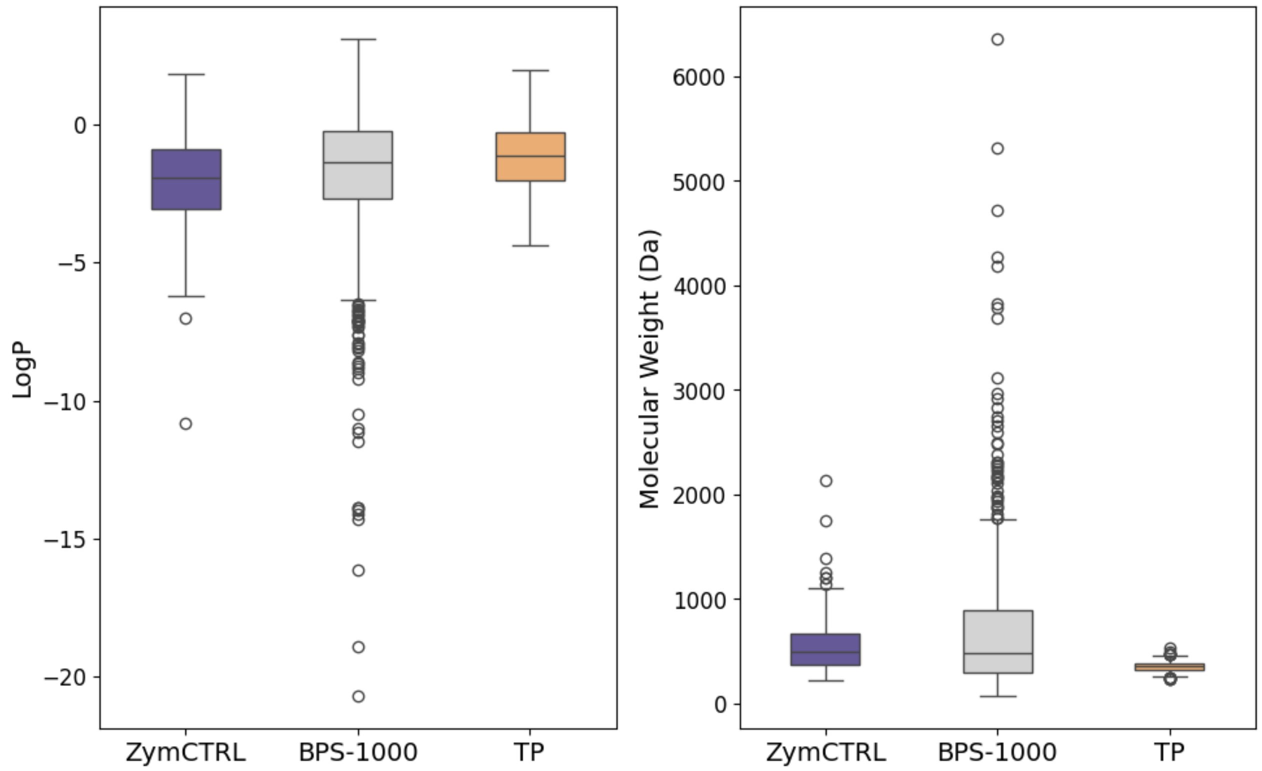

Figure S1. Boxplot distribution inclusive statistical parameters of the LogP and the MW for the datasets ZymCTRL, coloured in violet, BPS-1000, coloured in light grey, and the TP, coloured in orange.

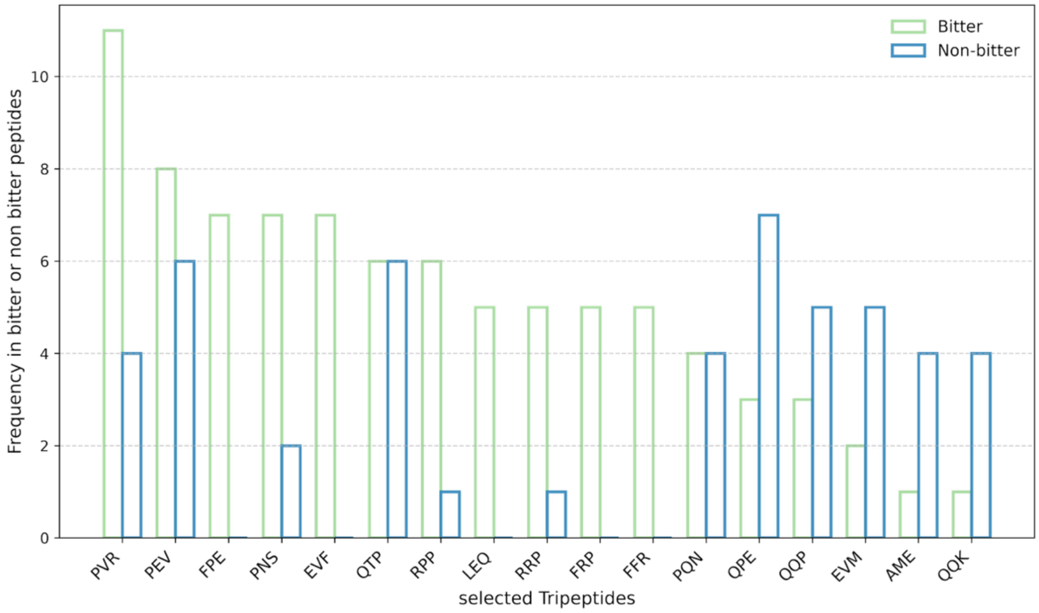

Figure S2. The frequency in absolute numbers of selected tripeptide sequences within the bitter and non-bitter dataset of the BPS-1000.

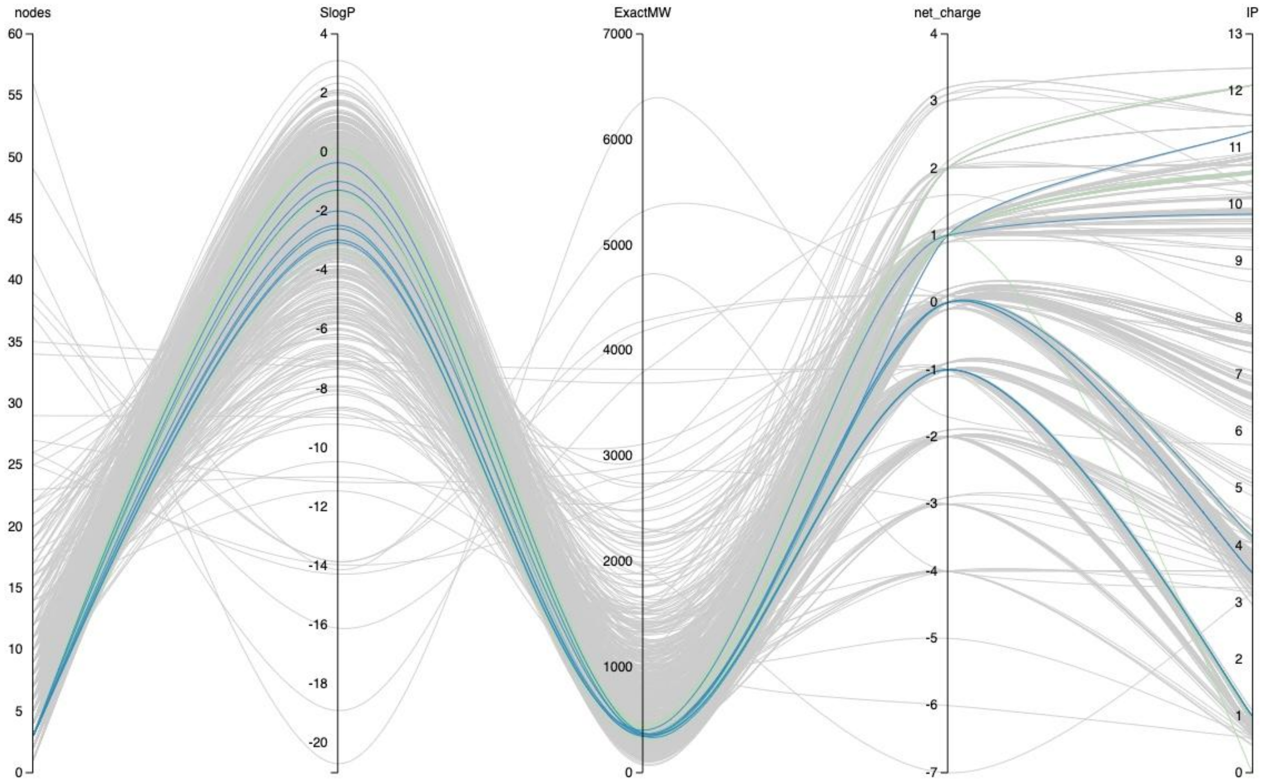

Figure S3. Line plot of the molecular descriptors: nodes (= number of amino acids), LogP(=SlogP), ExactMW (=Molecular weight in Da), net charge of peptides, IP (= isoelectronical point). The distribution of the BPS-1000 is shown in grey. The selected peptides are colored according to their bitterness prediction (green for bitter, blue for non-bitter).

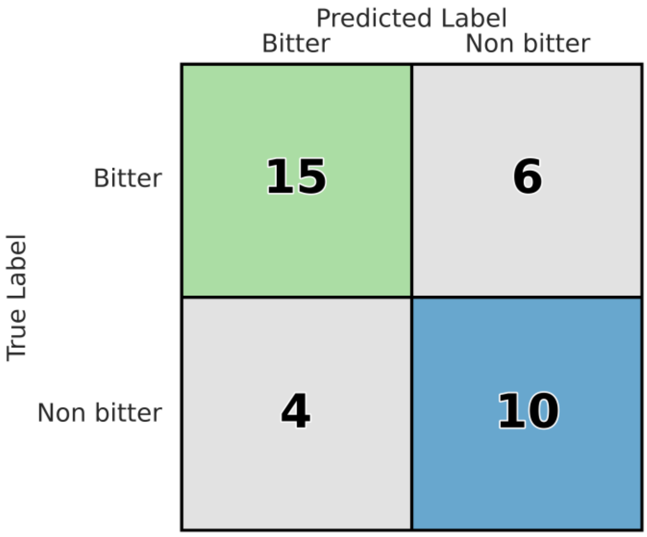

Figure S4. Sensitivity Analysis for Model Performance Under Worst-Case Misclassification. To provide a more conservative and transparent characterization of model performance, we conducted a sensitivity analysis in which the four peptides that yielded inconclusive sensory classification results (LDL, RPY, KALPQ, and KI) were treated as incorrect predictions. This worst-case scenario assumes that all ambiguous outcomes represent model failures. Under this assumption, model accuracy decreased from 0.80 to 0.71, precision from 0.93 to 0.79, and the F1-score from 0.83 to 0.75. In the fig, we report the confusion matrix reflecting this reassignment. While these adjusted metrics represent a conservative lower bound on model performance, they still demonstrate that the classifier retains meaningful discriminative capacity.

Table S1. Occurrence of peptides selected with ZymCTRL in BPS-1000 as part of longer peptides in this database

Peptide Sequence	Tate Prediction	Occurrence in BPS-1000 peptides
**RAPF**	bitter	No
**RQPF**	bitter	No
**KAP**	non-bitter	No
**KLP**	bitter	No
**GDFF**	bitter	No
**GPPG**	bitter	Present in 8 bitter peptides
**VAEQ**	non-bitter	Present in 1 bitter peptide
**TAEQ**	non-bitter	No
**LDL**	bitter	No
**RPY**	bitter	No
**KALPQ**	non-bitter	No
**KI**	bitter	Present in 7 bitter peptides
